# MCSNet: Channel Synergy-Based Human-Exoskeleton Interface With Surface Electromyogram

**DOI:** 10.3389/fnins.2021.704603

**Published:** 2021-11-17

**Authors:** Kecheng Shi, Rui Huang, Zhinan Peng, Fengjun Mu, Xiao Yang

**Affiliations:** ^1^School of Automation Engineering, University of Electronic Science and Technology of China, Chengdu, China; ^2^Center for Robotics, University of Electronic Science and Technology of China, Chengdu, China; ^3^Engineering Research Center of Human Robot Hybrid Intelligent Technologies and Systems, Ministry of Education, University of Electronic Science and Technology of China, Chengdu, China; ^4^Department of Orthopedics, Sichuan Provincial People's Hospital, University of Electronic Science and Technology of China, Chengdu, China

**Keywords:** human-robot interface, lower limb movement prediction, channel synergy-based network, exoskeleton, paraplegic patients, surface electromyography

## Abstract

The human–robot interface (HRI) based on biological signals can realize the natural interaction between human and robot. It has been widely used in exoskeleton robots recently to help predict the wearer's movement. Surface electromyography (sEMG)-based HRI has mature applications on the exoskeleton. However, the sEMG signals of paraplegic patients' lower limbs are weak, which means that most HRI based on lower limb sEMG signals cannot be applied to the exoskeleton. Few studies have explored the possibility of using upper limb sEMG signals to predict lower limb movement. In addition, most HRIs do not consider the contribution and synergy of sEMG signal channels. This paper proposes a human–exoskeleton interface based on upper limb sEMG signals to predict lower limb movements of paraplegic patients. The interface constructs an channel synergy-based network (MCSNet) to extract the contribution and synergy of different feature channels. An sEMG data acquisition experiment is designed to verify the effectiveness of MCSNet. The experimental results show that our method has a good movement prediction performance in both within-subject and cross-subject situations, reaching an accuracy of 94.51 and 80.75%, respectively. Furthermore, feature visualization and model ablation analysis show that the features extracted by MCSNet are physiologically interpretable.

## 1. Introduction

The development of artificial intelligence technology and wearable sensors has promoted the rise of human–robot interaction. As the core of human–robot interaction, an human–robot interface (HRI) enables direct communication with a robot via physical or biological signals, which has received widespread attention in the past decade (Simao et al., 2019; Fang et al., 2020). Exoskeleton is a typical application scenario of HRI, and some HRI based on physical signals, such as inertial measurement units or pressure signals, have been used in the walking-assistant exoskeleton to realize the movement prediction of patients with hemiplegia/paraplegia (Beil et al., 2018; Ding et al., 2020; Zhu et al., 2020a). In recent years, with the decoding of biological signals, HRI based on biological signals (such as electroencephalogram and electromyography) have been designed, opening up the possibility of realizing more natural and efficient movement predictions between human and exoskeleton (Suplino et al., 2019; Ortiz et al., 2020; Zhuang et al., 2021). For paraplegic patients, the loss of lower limb motor and sensory function makes the exoskeleton difficult to predict the patients' movement, and the previous work has not yet proposed a high-efficiency HRI specifically for paraplegic patients. Therefore, it is urgent to propose an HRI with high movement prediction accuracy for paraplegic patients.

Brain–computer interface (BCI) is an HRI based on electroencephalogram (EEG). It can directly obtain patients' motion intention from the EEG signal and without actual limb movement, so the BCI has been used to predict the movement of paraplegic patients (Tariq et al., 2018; Wang et al., 2018; Gu et al., 2020). The BCI consists of three main processing stages (Lotte et al., 2018): data collection and processing stage, where EEG data is recorded and preprocessed; feature extraction stage, where meaningful information is extracted from the EEG data; and classification stage, where a motion intention is interpreted from the data. The EEG signal's signal-to-noise ratio is low. It is susceptible to interference from the environment and the patient's own limb movement and mood, and the signal between different people is quite different (Rashid et al., 2020). The movement prediction accuracy of BCI is usually unstable, which is unacceptable for the exoskeleton movement assistance tasks of paraplegic patients.

Compared with the EEG signal, the sEMG signal has a higher signal-to-noise ratio and is less interfered with by external factors. Therefore, the sEMG-based human–robot interface (MHRI) has been earlier and more widely used in the walking-assistant exoskeleton (Kawamoto et al., 2003; Wang et al., 2021). The previous MHRI mostly used the sEMG signal of the lower limb muscles to predict movements. However, the sEMG signal of the lower limbs of paraplegic patients is weak or even no signal. So recent studies have attempted to use the sEMG signal of the upper body muscles to predict the lower limb movement (Villa-Parra et al., 2018). Similarly, MHRI also includes three stages of data collection and processing, feature extraction, and classification. Each stage relies on manual specifications. Many outstanding studies have shown that feature extraction is crucial for MHRI movement prediction, and it determines the upper limit of the prediction accuracy (Phinyomark et al., 2012; Samuel et al., 2018). Feature extraction often requires significant subject-matter expertise and a priori knowledge about the expected sEMG signal (Xiong et al., 2021). It is tough and time consuming to obtain an optimal feature set manually for different subjects.

Deep learning has largely alleviated the need for manual feature extraction, achieving state-of-the-art performance in fields such as computer vision and natural language processing (Hinton et al., 2012). In fact, deep convolutional neural networks (CNNs) can automatically extract appropriate features from the data. It has succeeded in many challenging image classification tasks (Huang et al., 2017; Jeyaraj and Nadar, 2019), surpassing methods that rely on handcrafted features (Hinton et al., 2012; Huang et al., 2017). Although most research still relies on handcrafted features, many recent works have explored the application of deep learning in MHRI (Allard et al., 2016; Cote-Allard et al., 2019; Jabbari et al., 2020). This kind of MHRI mostly combines long short-term memory networks (LSTM) and CNNs simply, ignoring the difference in contribution and synergy of sEMG feature channels of different subjects under the same movement. Moreover, most researchers do not pay much attention to whether the features extracted by CNNs have physiological significance.

In this paper, a channel synergy-based MHRI is proposed for lower limb movement prediction in paraplegic patients. It uses the sEMG signals of 12 upper limb muscles to predict the lower limb movements. The proposed movement prediction model uses LSTM, depthwise and separable convolutions to extract the spatiotemporal features of multi-channel sEMG signals, and introduces an attention module to extract the synergy of different sEMG feature channels. An sEMG data acquisition experiment is designed to verify the proposed channel synergy-based network (MCSNet). The experimental results verify that MCSNet's prediction accuracy is better than the traditional machine learning-based MHRI and two mainstream deep learning-based MHRI in both within-subject and cross-subject situations. Furthermore, we visualize the features extracted through MCSNet model and perform model ablation analysis. The analysis results show that the features proposed by MCSNet are physiologically interpretable.

In summary, the main contributions of this paper are shown as follows:

- A channel synergy-based MHRI is proposed for lower limb movement prediction of paraplegics. It uses the sEMG signals of upper limb to predict lower limb movements, and extracts the contribution, spatiotemporal, and synergy features among different sEMG channels, which improves the accuracy of lower limb movement prediction.
- This paper visualizes the features proposed by the MCSNet model and performs the model ablation analysis, and the results show that the features proposed by MCSNet are physiologically interpretable.

## 2. Related Works

Human–robot interfaces used to predict the movement of patients with damaged limb are mainly divided into BCI and MHRI.

### 2.1. BCI-Based Movement Prediction Related Work

The research of neuroengineering promotes the development of BCI, and it is mainly used in the field of medical rehabilitation to realize the perception of user intent. An entire BCI includes three main processing stages of data collection and processing, feature extraction, and classification (Lotte et al., 2018). Traditional BCI mainly extracts some manual normative time-domain, frequency-domain, and spatial domain features (Lee et al., 2019), and then uses machine learning methods to construct the mapping between features and different movements (Kaper et al., 2004; Wang et al., 2017). Wang et al. proposed a BCI based on support vector machine (SVM). It uses the common space pattern (CSP) model to extract the spatial features of the subject's motor imagery (MI) EEG signals, and uses the SVM model to realize the classification of lower limb movements (Wang et al., 2017).

Recent research has explored the application of deep learning in BCI (Tayeb et al., 2019; Tortora et al., 2020). Tayeb et al. used a CNN architecture to predict the movement of the raw MI EEG signals, achieving an accuracy of 84% (Tayeb et al., 2019). Tortora et al. proposed a gait pattern prediction method based on an LSTM architecture. This method uses the LSTM model to automatically extract and classify the timing features of the EEG signal (Tortora et al., 2020), which can achieve an accuracy of 92.8%. Considering the low signal-to-noise ratio of EEG signals, some research have tried to combine EEG with other signals to improve the movement prediction accuracy. Zhu et al. used the combination of EEG and electrooculogram (EOG) signals to realize the grasping and moving tasks of the robotic arm (Zhu et al., 2020b), with an average accuracy of 92.09%. BCI is unacceptable for the exoskeleton movement assistance tasks of paraplegic patients, because EEG signal is susceptible to interference from the environment and the patient's own limb movement and mood (Rashid et al., 2020).

### 2.2. MHRI-Based Movement Prediction Related Work

As the biological signal most relevant to exercise, sEMG has been applied to human–robot interaction for a long time, and the research on MHRI is particularly rich. According to the granularity of movement prediction, traditional MHRI can be divided into two categories, one is MHRI based on motion curve prediction, and the other is MHRI based on motion mode(movement) prediction. The former uses machine learning methods or Hill's musculoskeletal model to build a mapping between handcrafted features and joint angles/torques, which can achieve finer-grained movement prediction. Literature (Suplino et al., 2020) proposed an elbow joint angle estimation model based on a non-linear autoregressive with exogenous inputs neural network. This model can accurately predict the elbow joint's torque and angle during flexion and extension movement, with a mean square error within 7°. This kind of MHRI can only be predicted in one movement. The model involves many parameters and requires high quality of the sEMG signal, which is not suitable for the movement prediction of paraplegic patients.

The MHRI in the back is similar to BCI, which also includes three processing stages. Its main principle is using machine learning methods to map handcrafted features and movements (Afzal et al., 2017; Li et al., 2017; Cai et al., 2019; Kyeong et al., 2019; Tao et al., 2019). Cai et al. proposed an SVM-based upper limb movement prediction method (Cai et al., 2019), which uses the sEMG signal of the uninhibited upper limb muscle of the hemiplegic patient to predict the movement of the patient's shoulder and elbow joints, with an accuracy of 93.56%. Tao et al. proposed a multi-channel lower limb movement prediction method based on back propagation neural network, which can achieve an prediction accuracy of 93.6% in six lower limb movements such as the flexion movement of hip joint (Tao et al., 2019).

Deep learning can automatically extract the best feature set from sEMG signals. Many researchers have explored the application of deep learning in MHRI-based movement prediction methods (Allard et al., 2016; Cote-Allard et al., 2019; Jabbari et al., 2020). Allard et al. proposed a multi-layer CNN gesture prediction model based on sEMG for robot guidance tasks (Allard et al., 2016). The model automatically extracts the frequency domain features of different gesture movements through the CNN architecture, and the average accuracy of gesture prediction for 18 subjects is 93.14%. Considering the effectiveness of the LSTM architecture for timing feature extraction, Jabbari et al. proposed an ankle joint movement prediction model based on the CNN–LSTM architecture. The CNN and LSTM architectures were used to extract the spatial and temporal features of the sEMG signals, respectively, under different ankle joint movements (Jabbari et al., 2020), and the prediction accuracy of five ankle joint movements is 97.55%. Most deep learning-based MHRIs combine LSTM and CNNs simply to extract the timing or time-frequency features of the sEMG signal, but ignore the contribution and synergy differences of the sEMG feature channels of different subjects under the same movement. These are important features for different limb movements (d'Avella et al., 2003).

### 2.3. Application of HRI on Exoskeleton

As a tightly human–machine coupled system, the exoskeleton is a typical application scenario of HRI. The application of HRI on exoskeleton can be divided into movement prediction (Kyeong et al., 2019; Read et al., 2020) and state monitoring (Bae et al., 2019). Movement prediction is to help the exoskeleton recognize the wearer's motion intention and realize natural human–exoskeleton interaction. An HRI based on the wearer's upper limb inertial measurement unit signal and crutches pressure signal was applied to the Ekso exoskeleton (Read et al., 2020). It helps the exoskeleton realize the prediction of standing and walking movements. Kyeong et al. proposed a hybrid HRI based on the wearer's lower limb sEMG signals and the sole pressure signals (Kyeong et al., 2019), achieving the prediction of the gait cycle. HRI based on state monitoring is to help observe the changes in the wearer's physiological state when using the exoskeleton. Bae et al. designed an MHRI for their wrist-rehabilitation exoskeleton robot (Bae et al., 2019). It can monitor whether the wearer has spasticity during the exoskeleton assistance task.

Our work is mainly based on the lower limb movement prediction of the walking-assistant exoskeleton for paraplegia patients. It is most closely related to the MHRI based on deep learning, which uses CNN and LSTM architecture to extract the sEMG signal features of different lower limb movements. In contrast to deep learning-based MHRI, this paper propose a channel synergy-based MHRI, which extracts the contribution and synergy of the sEMG feature channel. Its performance is better than traditional machine learning-based MHRI and two mainstream deep learning-based MHRI.

## 3. Methods

This section presents the methodology details of the proposed movement prediction model. Section 3.1 describes the overall architecture of the MCSNet model. In section 3.2, we introduce seven traditional MHRIs and two mainstream deep learning-based MHRIs, which are used to compare to the MCSNet model.

### 3.1. Description of the MCSNet Model

**Figure 1:**
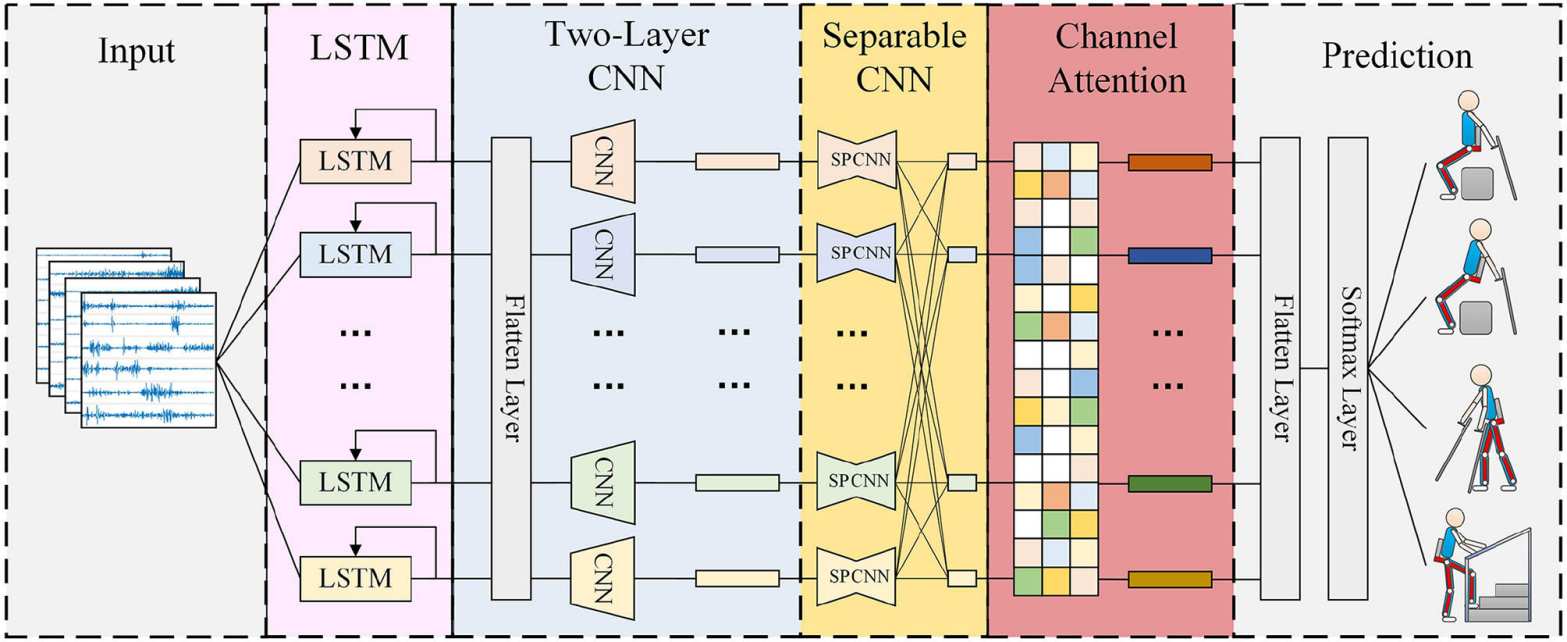
Overall architecture of the MCSNet model. Lines denote the convolutional kernel connectivity between inputs and outputs (called feature maps). The network starts with a channel-by-channel long short-term memory networks (LSTM) (second column) to learn the timing feature, then uses a two-layer convolution (third column) to learn different spatiotemporal features. The separable convolution (fourth column) is a combination of a depthwise convolution followed by a pointwise convolution, which can explicitly decouple the relationship within and across feature maps and learns the synergy feature of surface electromyography (sEMG).

Figure 1 visualizes the proposed MCSNet model. The entire model architecture consists of three parts. The first part is data input, input the processed sEMG data; the second part is feature extraction, which mainly contains four blocks, each block establishes the connection between the feature channels of the sEMG signal in different dimensions; the third part is movement classification/prediction, which classifies the extracted features. This section mainly describes the feature extraction part, because it is the core of the entire model. For sEMG trials, it was collected at a 1,500 Hz sampling rate, having *C* channels and *T* time samples.

- sEMG is a kind of non-stationary time series data. For movement prediction, extracting more timing features is the basic requirement to improve accuracy. In block 1, for each input sEMG sample segment (size *C* × 300, multiple shown in Figure 1), we performed a channel-by-channel LSTM step to extract the timing features of different signal channels. Since the deepening of the LSTM layers will cause over-fitting, we found this phenomenon is more serious for sEMG data during the experiment, so we choose to use a single-layer LSTM as the timing feature extraction block. In this process, we define the *k*th sEMG channel signal as
  (1) $$\begin{array}{r} F_{sEMG}^{k},\left(k=1,...,C\right) \end{array}$$
  which *k* indicates the serial number of the channel. In order to better describe the relationship between the LSTM block and the sEMG feature channel, a more fine-grained channel-by-channel representation is used. The operation with LSTM block is defined as follows:
  (2) $$\begin{array}{r} F_{temp}^{k}=N_{lstm}^{k}\left(F_{sEMG}^{k}\right), \end{array}$$
  In Equation (2), each of the sEMG signal channels is used to generate its timing feature independently, the timing feature from all the channels will be contacted into *F*_*temp*_, which size is C*L, *L* represents the length of input signal's sample. Since the input feature channel $F_{sEMG}^{k},\left(k=1,...,C/2\right)$ and $F_{sEMG}^{k+C/2},\left(k=1,...,C/2\right)$ in our data acquisition process is opposite the left and right symmetrical relationships on the muscle blocks in the acquisition, the muscles of the symmetry position have similar behavior patterns when the subjects are under various movements, so we use the LSTM units with shared weights used in the corresponding channel.
- In block 2, we perform two convolutional steps in sequence. First, we fit *F*_1_ 2D convolution filters with a size of (1, 65) and output F1 feature maps containing different timing information. We then use a depthwise convolution of size (*C*, 1) (Chollet, 2017) to extract spatial features for every channel. This operation provides a direct way to learn spatial filters for different timing information, which can effectively extract different timing and spatial features. The depth parameter *D* represents the number of spatial filters to be learned for each time series feature map (*D* = 1 is shown in Figure 1 for illustration purposes). In this block, *F*_*temp*_ is transformed with the first convolution layer as follows:
  (3) $$\begin{array}{r} F_{conv}=N_{conv}\left(F_{temp}\right), \end{array}$$
  (4) $$\begin{array}{r} F_{d-conv}=N_{d-conv}\left(F_{conv}\right), \end{array}$$
  In Equations (3) and (4), the size of *F*_*conv*_ and *F*_*d*−*conv*_ is *F*_1_*C*L and (D*F_1_)*1*L, respectively.
- In block 3, we use a separable convolution, a depthwise convolution of size (1, 15) followed by *F*_2_ pointwise convolutions of size (1, 1). The separable convolutions first learn the kernel of each spatiotemporal feature map individually, then optimally merge the outputs afterward, which can explicitly decouple the relationship within and across feature maps. This operation separates the learning of spatiotemporal features from the combination of optimal features, which is very effective for sEMG signals. Because sEMG signals have different synergy between channels when performing different movements (muscle synergy effect, d'Avella et al., 2003), this is similar to a synergy feature, which the separable convolutions can extract. Because the padding is used in the first stage of separable convolution, and the pixel-wised convolution will not change the size of the feature, the output *F*_*sep*−*conv*_ has the same size as *F*_*d*−*conv*_.
- For block 4, we introduced a channel attention module. This operation learns the weights of different synergy features, which can effectively associate movements with the most relevant synergy features and improve the movement prediction accuracy. Moreover, there are differences in the feature contributions of sEMG channels in different subjects under the same movement (muscle compensatory behavior, d'Avella et al., 2006), which will amplify the differences in the synergy feature of different subjects under the same movement. The channel attention module can learn different weights for different subjects to deal with the differences in synergy features, thereby improving the robustness of the entire movement prediction model. The operation of this block can be described as:
  (5) $$\begin{array}{l} W_{channel}(F_{sep-conv})=\sigma (MLP(AvgPool(F_{sep-conv}))\\ \;+MLP(MaxPool(F_{sep-conv}))), \end{array}$$
  (6) $$\begin{array}{r} F=W_{channel}\left(F_{sep-conv}\right)⊗F_{sep-conv}, \end{array}$$

We input the generated attention-based spatiotemporal features into the movement classification/prediction part. As shown in Figure 1, the extracted features first perform a Flatten layer step, and then pass directly to a softmax classification with *N* units, where *N* is the number of classes in the data. The entire model architecture uses the cross-entropy loss function to optimize the parameters, and input 10 sEMG samples with time-sequence everytime.

### 3.2. Comparison With Other MHRI Movement Prediction Approaches

#### 3.2.1. Comparison With Traditional MHRI Movement Prediction Approaches

We compared the performance of MCSNet with seven traditional MHRI based on handcrafted features and machine learning models in lower limb movement prediction. In the selection of features, referring to the research conclusions of time domain and frequency domain features in the literature (Phinyomark et al., 2012) and four commonly used feature sets (Englehart and Hudgins, 2003) (Phinyomark et al., 2013), we finally select the feature of Mean Absolute Value (MAV), WaveLength (WL), Zero Crossings (ZC), 6-order AutoRegressive coefficient (6-AR), and average Power Spectral Density (PSD). Furthermore, we choose Linear Discriminant Analysis (LDA), Decision Tree (DT), Naive Bayes (BES), Linear Kernel-based Support Vector Machine (LSVM), Radial Basis Function-based Support Vector Machine (RBFSVM), K Nearest Neighbor (KNN), and Artificial Neural Network (ANN) as the classification/prediction model. We use MATLAB's Classification Learner Toolbox and Neural Net Pattern Recognition Toolbox to implement these models. The hyperparameter settings of each model are shown in Table 1.

**Table 1:** Parameter list of traditional MHRI movement prediction approaches.

**Method**	**Hyperparameter and model detail setting**
LDA	Covariance structure: full rank (for within-subject), diagonal (for cross-subject)
DT	Maximum fission number: 100
BES	Kernel: Radial Basis Function,
LSVM	Kernel: linear, C = 1, Multiple classification method: OVO
RBFSVM	Kernel: RBF, C = 1.9, Multiple classification method: OVO
KNN	Number of neighboring points: 1, Metric function: mahalanobis distance function
ANN	Number of hidden unit: 28

#### 3.2.2. Comparison With Deep Learning-Based MHRI Movement Prediction Approaches

In deep learning, we compared the performance of MCSNet with two-layers CNN (TCNN) and CNN-LSTM models. The TCNN architecture consists of two convolutional layers and a softmax layer which is for classification. The CNN-LSTM architecture includes two LSTM layers, three convolutional layers, and a softmax layer. We implemented these models in PyTorch. For specific details of the model, see https://github.com/mufengjun260/MCSNet.

In general, the most significant difference between MCSNet and traditional MHRI movement prediction approaches is the feature extraction method, and the most significant difference from other deep learning-based movement prediction methods is the network architecture. By comparing with other methods, we can prove the effectiveness of the feature extraction architecture we designed.

## 4. Experiments and Results

In this part, an sEMG signal acquisition experiment based on upper limb muscles is designed to verify the effectiveness of the method proposed in this paper. Section 4.1 describes the process of the acquisition experiment and the process of data preprocessing. Section 4.2 gives the implementation details of model training. In section 4.3, we show the MCSNet movement prediction model results and compare MCSNet with other movement prediction models in the case of within-subject and cross-subject. Section 4.4 explains the results of MCSNet model ablation analysis and feature visualization.

### 4.1. sEMG Data Acquisition Experiment

A total of 8 healthy subjects were invited to participate in the experiment. Each subject completed four lower limb movements of standing, sitting, walking, and going up stairs while wearing the AIDER exoskeleton. During this period, the sEMG signals of the subjects' upper limbs were collected.

**Figure 2:**
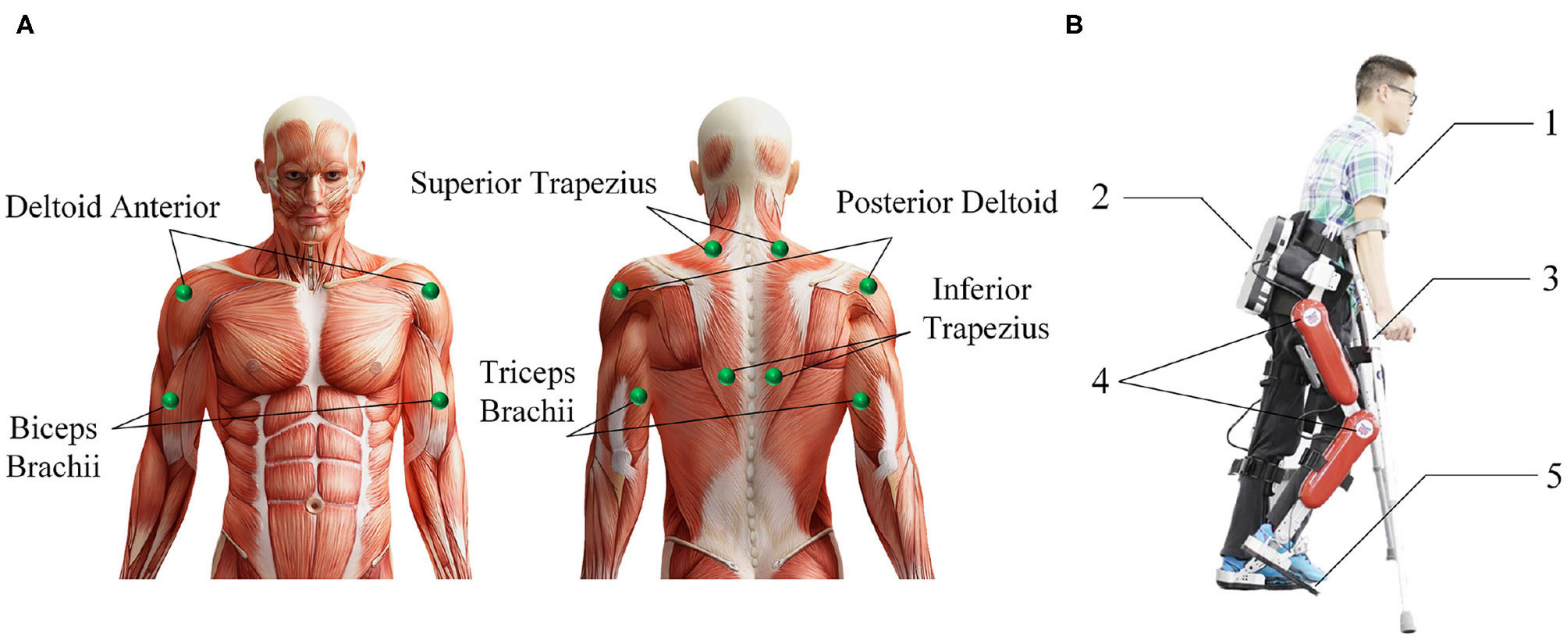
Introduction of the muscle used in the surface electromyography (sEMG) data acquisition experiment and the AssisIve DEvice for paRaplegic patient (AIDER) exoskeleton. **(A)** The upper limb muscle used in sEMG data acquisition experiment. **(B)** The AIDER exoskeleton is designed for walking assistance of paraplegic patients, and it can help the paraplegic patient complete some ADL movements such as sitting, standing, walking, and going upstairs movement. 1: The subject; 2: the embedded computer and IMU; 3: the crutches; 4: DC servo motors; 5: intelligent shoes with plantar pressure sensors inside.

**Figure 3:**
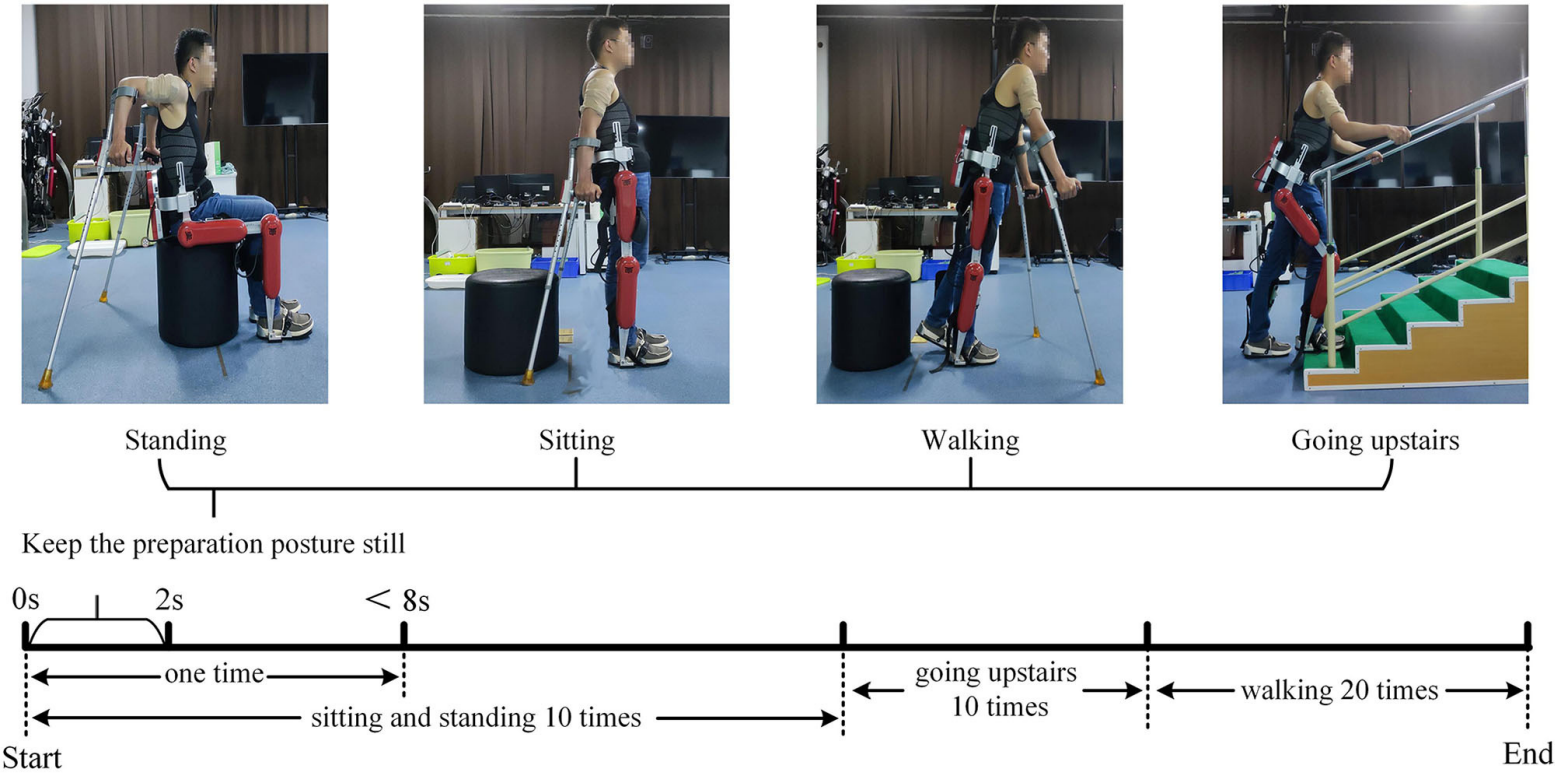
Schematic diagram of surface electromyography (sEMG) data acquisition experiment. The upper part is the preparation posture of the four lower limb movements. We fixed the sEMG acquisition electrode with an elastic bandage to prevent the acquisition electrode from falling off during the experiment. The lower part is the schematic diagram of the experimental acquisition process.

1. ***Participants***: The eight subjects (7 males, one female) had an average age of 26 years, a height between 165 and 185 *cm*, and a weight between 59 and 82 *kg*. All subjects can independently use the AIDER exoskeleton to complete the lower limb movements involved in the experiment, and are in good physical condition with no injuries to the arm. Before the experiment, each subject had been explained the contents of the experiment and signed an informed consent form. This experiment was approved by the Research Ethics Committee of the University of Electronic Science and Technology of China.
2. ***Procedures***: Before the experiment, record the relevant physical parameters of the subject, inform the experimental procedure to the subject, and let the subject use crutches to freely practice the four lower limbs movements of standing, sitting, walking, and going upstairs while wearing the AIDER exoskeleton for 30 *min*. Then paste sEMG acquisition electrodes on the 12 muscles of the subject's left and right upper limbs, including the deltoid anterior, biceps, and superior trapezius muscles (as shown in Figure 2). Before pasting, wipe the corresponding muscles with alcohol cotton and remove the surface hair with a hair removal knife. The subject puts on the AIDER exoskeleton (Wang et al., 2019), supports the crutches with both hands, stands in the designated position, and completes the sitting, standing, and going upstairs movements 10 times after hearing the instructions, and then completes walking movement 20 times (a complete gait cycle is one time). Each movement is completed within 8 s, all subjects are required to perform the specified movements without using their legs as much as possible to ensure that the collected upper limb sEMG signals are close to the paraplegic patients. After the movement starts, the subject maintains the lower limb movement preparation posture for 2 *s* (see Figure 3) and then controls the AIDER exoskeleton to complete the corresponding lower limb movement. Throughout the experiment, the camera is turned on to record, and myoMUSCLE (an sEMG acquisition device, Scottsdale, American) is used to collect the sEMG signals of the upper limbs.
3. ***Data Processing***: myoMUSCLE (1,500 Hz) collects the upper limb sEMG signal data of each lower limb movement of the subject throughout the whole process. After obtaining the sEMG data, a 50 Hz notch filter is used to remove the power frequency interference of the current, and a 10–450 Hz bandpass filter is used to retain the effective information of the sEMG signal. Since our application is lower limb movement prediction, we only intercept the sEMG data during the movement preparation period (the period when keeping the preparation posture still). In addition, to achieve continuous movement prediction of lower limb, this paper uses 200 *ms* (including 300-time series data) as a time window to segment the sEMG signal, and the movement step of the time window is 100-time series data.

### 4.2. Implementation Details

After preprocessing the sEMG data, for the traditional MHRI movement prediction model, use the relevant formula to calculate the features mentioned in section 3.2.1, and then input the features into the Classification Learner Toolbox and Neural Net Pattern Recognition Toolbox to train the prediction model. For the problem of imbalance in the number of samples between movements, we apply a movement class-weight to the loss function. The class-weight we apply is the inverse of the proportion in the training data, with the majority movement class set to 1.

MCSNet and the deep learning-based MHRI movement prediction models are implemented using the PyTorch library (Paszke et al., 2017). In MCSNet, both LSTM's output and hidden unit are of dimension 300, and the network's hyperparameters (*D*, *F*1, *L*) is set to (2, 12, 300). The model with TCNN uses the same dimension as the MCSNet's CNN layers, and the CNN-LSTM model enlarged the deepness of MCSNet's LSTM block, it uses a two-layer LSTM network architecture. Exponential linear units (ELU) (Clevert et al., 2015) are used to introduce the non-linearity of each convolutional layer. To train ours and other deep learning-based models, we use the Adam optimizer to optimize the model's parameters, with default setting described in (Kingma and Ba, 2014) to minimize the categorical cross-entropy loss function. We run 1,000 training iterations (epochs) and perform validation stopping, saving the model weights, which produce the lowest validation set loss. All models are trained on NVIDIA RTX2080Ti, with CUDA10.1 and cuDNN V7.6. Our code implementation can be found in https://github.com/mufengjun260/MCSNet.

### 4.3. Experiments Result

We compared the performance of the proposed MCSNet model with other MHRIs in movement classification/prediction in both the within-subject and cross-subject situations.

#### 4.3.1. Within-Subject Classification

For within-subject, we divide the data of the same subject according to a ratio of 7:3 and then use 70% of the data to train the model for that subject. Four-fold cross-validation is used to avoid the phenomenon of model overfitting. Simultaneously, repeated-measures analysis of variance (ANOVA) is used to test the results statistically (using the number of subjects and the classification model as factors, and the model classification/prediction result (accuracy) as the response variable).

**Figure 4:**
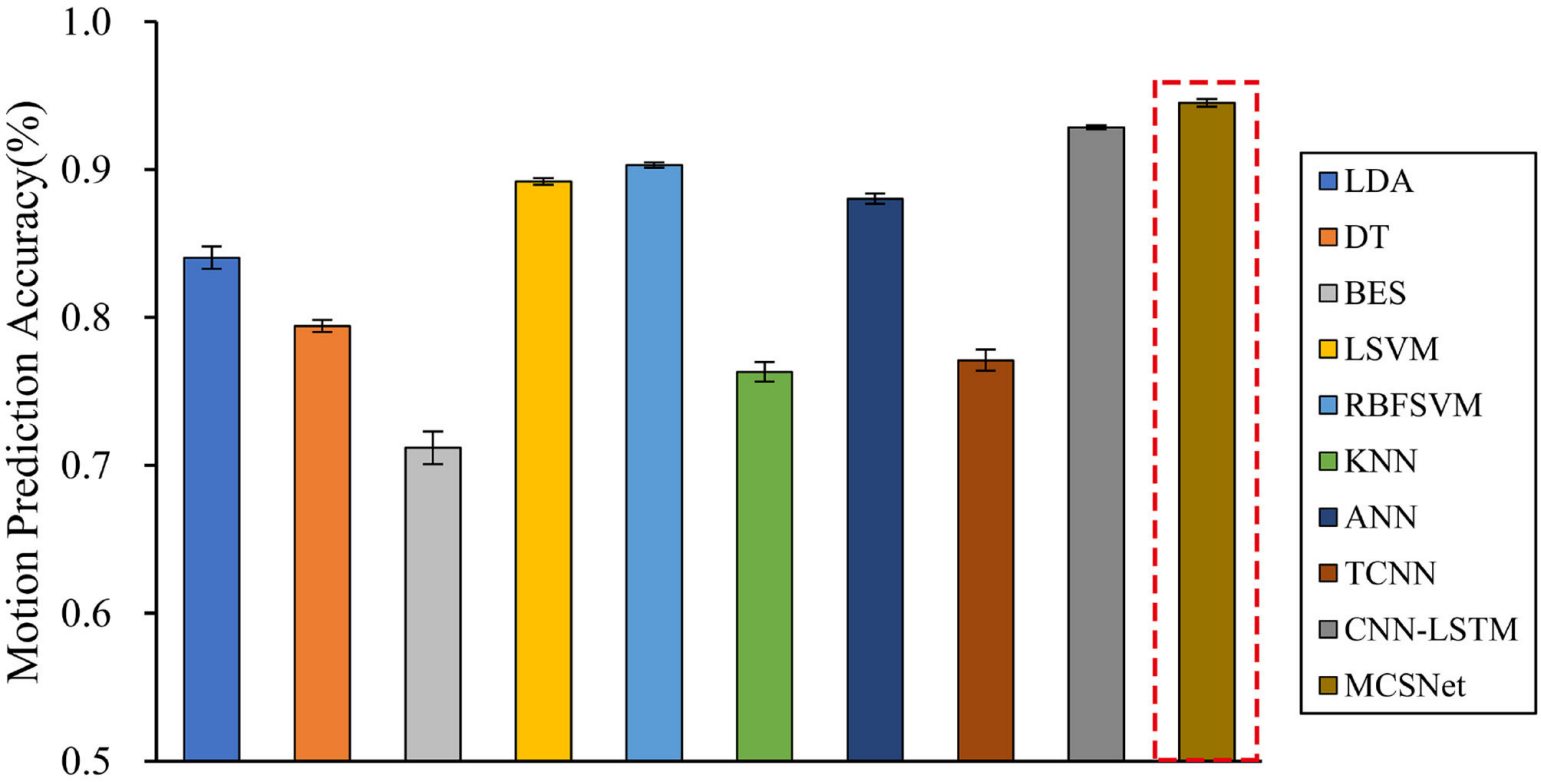
Within-subject movement prediction performance, four-fold cross-validation is used to avoid the phenomenon of model overfitting, averaged over all folds and all subjects. Error bars denote two standard errors of the mean.

We compare the performance of both traditional machine learning-based MHRI movement prediction models (LDA, DT, BES, LSVM, RBFSVM, KNN, and ANN) and deep learning-based MHRI movement prediction models (TCNN and CNN-LSTM) with MCSNet. Within-subject results across all models are shown in Figure 4. It can be observed that, across the average lower limb movement prediction accuracy of 7 subjects, MCSNet outperforms traditional machine learning-based and deep learning-based MHRI models. But there is no significant statistical difference (*P* > 0.05). Among the traditional MHRI movement prediction models, the RBFSVM model has the highest average accuracy of 7 subjects, reaching 90.31%. It is consistent with the conclusions obtained in previous work (Ceseracciu et al., 2010). Table 2 shows the prediction accuracy of each subject under different MHRI movement prediction models. It can be found that the same movement prediction model has a large difference in the accuracy for different subjects (especially the traditional MHRI movement prediction model). In contrast, MCSNet has a high accuracy rate of lower limb movement prediction for all subjects, and the accuracy rate is evenly distributed. It means that MCSNet can effectively extract each subject's lower limb movement feature, thereby achieving good movement prediction.

**Table 2:** Within-subject movement prediction performance (test set ACC).

	**Traditional machine learning-based MHRI**							**Deep learning-based MHRI**		
**Subject**	**LDA**	**DT**	**BES**	**LSVM**	**RBFSVM**	**KNN**	**ANN**	**TCNN**	**CNN-LSTM**	**MCSNet (ours)**
1	0.9200	0.7520	0.8496	0.9451	0.9504	0.8387	0.9315	0.8377	0.9570	**0.9928**
2	0.8731	0.8097	0.7718	0.9026	0.9159	0.8000	0.9008	0.5849	0.9034	**0.9295**
3	0.7105	0.8724	0.7852	0.8146	0.8503	0.6018	0.7590	0.7722	0.9089	**0.9772**
4	0.7888	0.6630	0.6818	0.8594	0.8526	0.7294	0.8428	0.7543	0.9075	**0.9513**
5	0.7430	0.7962	0.4937	0.8675	0.8911	0.7091	0.8828	0.8525	**0.9434**	0.9212
6	0.8872	0.8188	0.6747	**0.8936**	0.8927	0.8358	0.8923	0.8373	0.8844	0.8437
7	0.9600	0.8467	0.7263	0.9602	0.9687	0.8261	0.9523	0.7576	0.9960	**1.0000**
Average ACC	0.8404	0.7941	0.7119	0.8918	0.9031	0.7630	0.8802	0.7709	0.9287	**0.9451**

*Bold means the highest prediction accuracy rate of the subject corresponding to the row*.

#### 4.3.2. Cross-Subject Classification

In the case of cross-subject, we randomly selected the data of three subjects to train the model and selected the data of two subjects as the validation set. The whole process is repeated ten times, producing ten different folds.

**Figure 5:**
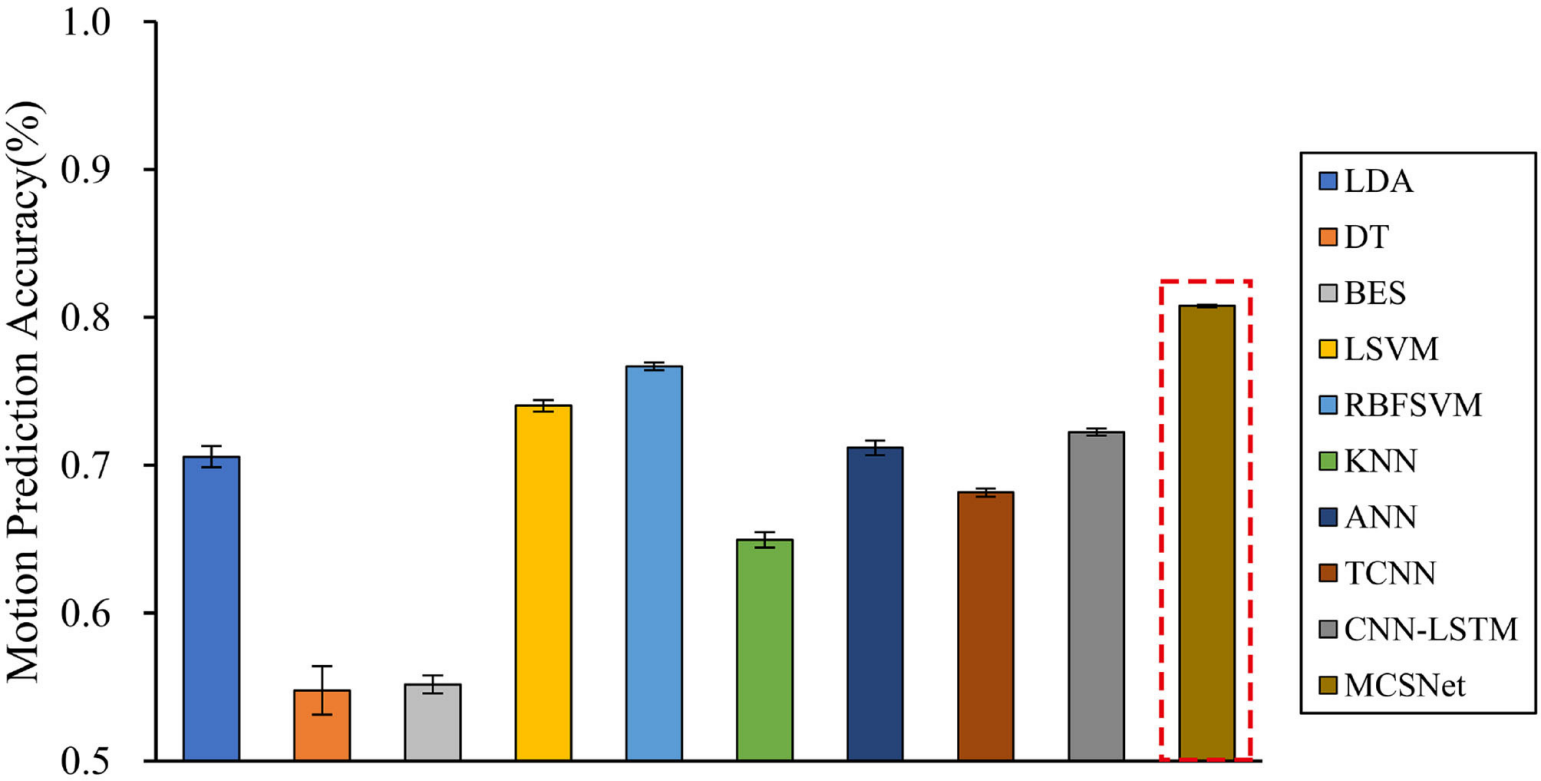
Cross-subject movement prediction performance, averaged over all folds. Error bars denote two standard errors of the mean.

Cross-subject prediction results across all models are shown in Figure 5. It can be seen that the traditional and deep learning-based MHRI movement prediction models have poor performance in the cross-subject situation, with an average accuracy rate of about 70%. However, the MCSNet model proposed in this paper can still achieve an accuracy of 80.25% in lower limb movement prediction, which has a significant statistical difference (*P* < 0.05). This result shows that the MCSNet model proposed in this paper can extract the deep common features of different subjects under the same lower limb movement. The model has good robustness.

### 4.4. MCSNet Feature Explainability

**Figure 6:**
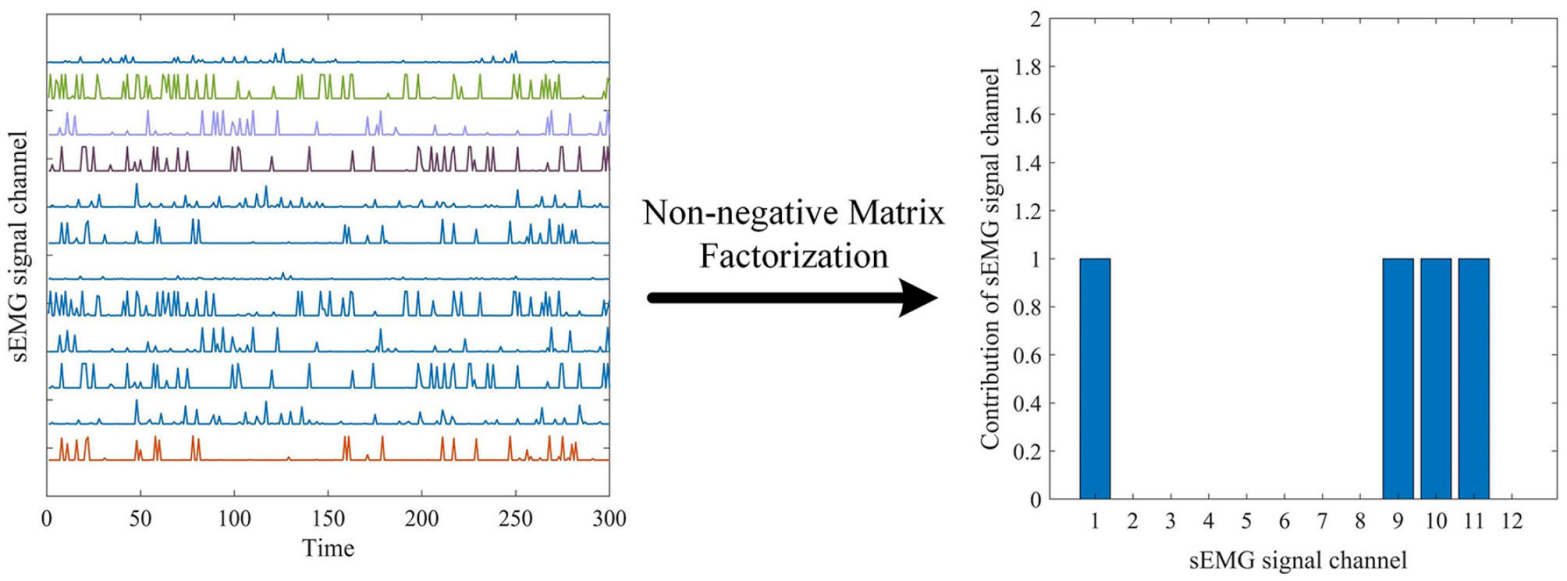
The average output of all surface electromyography (sEMG) signal samples about the sitting movement for subject 7, and non-negative matrix factorization method id used to find the synergy channels.

The development of methods for enabling feature explain-ability from deep neural networks has gradually become the focus of attention over the past few years, and has been proposed as an essential component of a robust model validation procedure, to ensure that the classification performance is being driven by relevant features as opposed to noise in the data (Ancona et al., 2017; Montavon et al., 2018). This paper uses data information flow tracking to understand the features proposed by the MCSNet model. Figure 6 shows the average output of all sEMG signal samples about the sitting movement for subject 7. Using the non-negative matrix factorization method, we can intuitively see that the sEMG channel 1, 9, 10, 11 are the main contribution channels for subject 7 to complete the sitting movement (i.e., the muscles corresponding to the channel 1, 9, 10, and 11 assume the main synergistic effect in the sitting movement) (d'Avella et al., 2003). Muscle synergy is an important physiological characteristic for humans to complete different movements. In order to explore whether the MCSNet network can reflect muscle synergy, we extracted the feature output and channel weights of each layer of MCSNet, and realized the information flow tracking of sEMG data through non-negative matrix factorization and weight screening.

**Figure 7:**
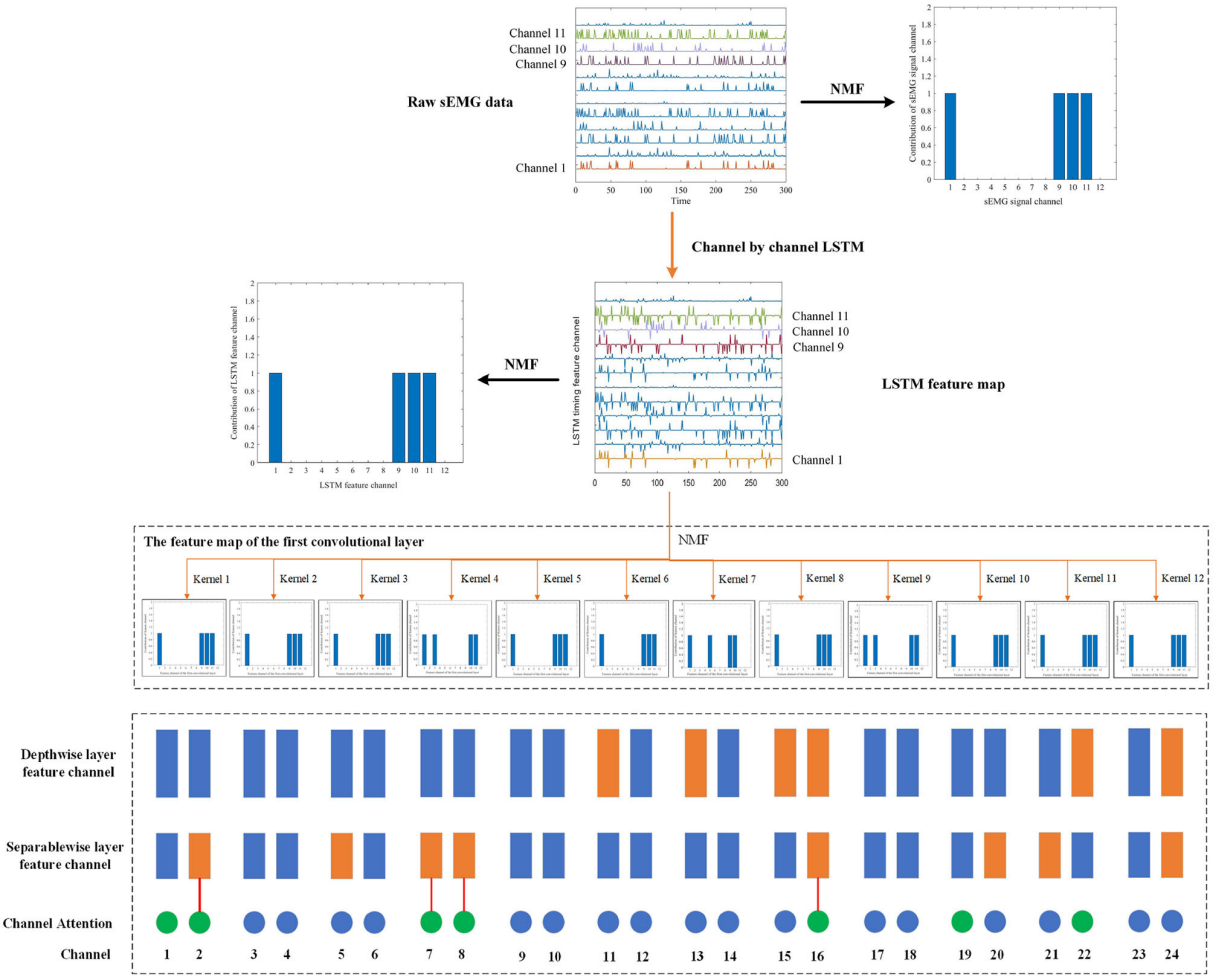
We visualized the synergy characteristics flow of surface electromyography (sEMG) in the sitting movement of subject 7 in the within-subject situation. The figure shows the flow of synergy characteristics in different feature channels of the MCSNet model (orange lines and rectangles). The blue rectangles represent the feature channels of the depthwise and separablewise network layers. The circle represents the weight channel of the attention layer, and the green circle means the channel with a large weight. We found that the channel with a large attention layer weight is basically the same as the channel of the synergy characteristics flow direction. It can be considered that MCSNet can extract the synergy characteristics of the muscle.

We performed non-negative matrix decomposition on the output of LSTM and the first convolutional layer, as shown in Figure 7. It can be observed that the main contribution channels of the features extracted by the LSTM and the first convolutional layer are still the channel 1, 9, 10, and 11, which means that the timing features currently extracted by MCSNet mainly come from the sEMG channel 1, 9, 10, and 11, and the synergy characteristics of these four channels are also included. The depthwise convolutional layer's function is to combine different timing feature channels, and then extract different spatiotemporal features. We analyzed the channel weights of the depthwise convolutional layer and focused on the spatiotemporal feature channels, which have a large weight for channel 1, 9, 10, and 11. Because these spatiotemporal feature channels are the main flow direction of the synergy characteristics. The results showed that the synergy characteristics are mainly contained in the spatiotemporal feature channels 11, 13, 15, 16, 22, and 24. In the same way, we analyzed the channel weights of the separable convolutional layer and compared the channels, which the synergy characteristics mainly flow, with the important channels learned by the attention mechanism. The results show that the channels selected by the two are basically the same (as shown in Figure 7). It means that the features extracted by MCSNet can reflect the synergy of muscles.

In addition, we performed a model ablation analysis on MCSNet under the cross-subject situation, removing depthwise, sparablewise, and attention network structure layers in turn and observing the changes in the prediction performance of the MCSNet model. According to the results in Table 3, removing any network structure layer will significantly reduce the prediction performance of the MCSNet model, which shows that each layer of the MCSNet model plays an essential role in the final prediction results.

**Table 3:** The result of model ablation analysis.

**Layer removed**	**Test set ACC**
Depthwise layer	0.7258
Sparablewise layer	0.7241
Attention layer	0.7187
None	0.8075

## 5. Conclusions

In this paper, a channel synergy-based human–exoskeleton interface is proposed for lower limb movement prediction in paraplegic patients. It uses the sEMG signals of 12 upper limb muscles as input signals, which can avoid the problem of weak sEMG signals in the lower limbs of paraplegic patients. The interface constructs an channel synergy-based network (MCSNet), it uses LSTM, depthwise, and separable convolutions to extract the spatiotemporal features of multi-channel sEMG signals, and introduces an attention module to extract the synergy of different sEMG feature channels. An sEMG acquisition experiment is designed to verify the effectiveness of the MCSNet model. The results show that MCSNet has a good movement prediction performance in both within-subject and cross-subject situations. Furthermore, feature visualization and the model ablation analysis of MCSNet is performed, the result show that the features extracted by MCSNet are physiologically interpretable. In the future, we consider applying the proposed human–exoskeleton interface to an actual exoskeleton platform. In addition, we will focus on multi-modal movement prediction based on sEMG and EEG.

## Data Availability Statement

The datasets presented in this study can be found in online repositories. The names of the repository/repositories and accession number(s) can be found below: https://github.com/mufengjun260/MCSNet.

## Ethics Statement

The studies involving human participants were reviewed and approved by Ethics Committee of University of Electronic Science and Technology of China. The patients/participants provided their written informed consent to participate in this study. Written informed consent was obtained from the individual(s) for the publication of any potentially identifiable images or data included in this article.

## Author Contributions

KS designed the movement prediction model, performed the experiments, and drafted the manuscript. RH and FM participated in the design of the movement prediction model and assisted in the manuscript writing. ZP and XY guided writing paper and doing experiments. All authors contributed to the article and approved the submitted version.

## Funding

This work was supported by the National Key Research and Development Program of China (No. 2018AAA0102504), the National Natural Science Foundation of China (NSFC) (No. 62003073), the Sichuan Science and Technology Program (Nos. 2021YFG0184, 2020YFSY0012, and 2018GZDZX0037), and the Research Foundation of Sichuan Provincial People's Hospital (No. 2021LY12).

## Conflict of Interest

The authors declare that the research was conducted in the absence of any commercial or financial relationships that could be construed as a potential conflict of interest.

## Publisher's Note

All claims expressed in this article are solely those of the authors and do not necessarily represent those of their affiliated organizations, or those of the publisher, the editors and the reviewers. Any product that may be evaluated in this article, or claim that may be made by its manufacturer, is not guaranteed or endorsed by the publisher.
